# CRYSTMET—The NRCC Metals Crystallographic Data File

**DOI:** 10.6028/jres.101.021

**Published:** 1996

**Authors:** Gordon H. Wood, John R. Rodgers, S. Roger Gough, Pierre Villars

**Affiliations:** Canadian Scientific Numeric Database Service, National Research Council Canada, Ottawa, Canada, K1A 0S2; Materials Phases Data Bank (MPDS), Vitznau, Switzerland

**Keywords:** alloys, computer-based, critically evaluated data, crystallographic data, data, intermetallics, machine-readable, metals, minerals, numeric data, search system, structural data

## Abstract

CRYSTMET is a computer-readable database of critically evaluated crystallographic data for metals (including alloys, intermetallics and minerals) accompanied by pertinent chemical, physical and bibliographic information. It currently contains about 60 000 entries and covers the literature exhaustively from 1913. Scientific editing of the abstracted entries, consisting of numerous automated and manual checks, is done to ensure consistency with related, previously published studies, to assign structure types where necessary and to help guarantee the accuracy of the data and related information. Analyses of the entries and their distribution across key journals as a function of time show interesting trends in the complexity of the compounds studied as well as in the elements they contain. Two applications of CRYSTMET are the identification of unknowns and the prediction of properties of materials. CRYSTMET is available either online or via license of a private copy from the Canadian Scientific Numeric Database Service (CAN/SND). The indexed online search and analysis system is easy and economical to use yet fast and powerful. Development of a new system is under way combining the capabilities of ORACLE with the flexibility of a modern interface based on the Netscape browsing tool.

## 1. Introduction

CRYSTMET, the National Research Council of Canada Metals Crystallographic Data File, is a computer-readable database of critically evaluated crystallographic data for metals (including alloys, intermetallics and minerals) accompanied by pertinent chemical, physical and bibliographic information. D. T. Cromer and A. C. Larson began compiling the database at Los Alamos in 1960. At the suggestion of W. B. Pearson, A. C. Larson brought the compilation to Canada in 1974 where it was further developed and extended at Ottawa by L. D. Calvert. CRYSTMET is now maintained and distributed by the Canadian Scientific Numeric Database Service (CAN/SND) of the National Research Council of Canada (NRCC) [1]. Since 1988, the Materials Phases Data Bank (MPDS) has been vitally involved in the production of CRYSTMET.

The coverage of CRYSTMET complements that of the three other major structural databases [2]: organics and organometallics are covered by the Cambridge Structural Database (CSD, Cambridge Crystallographic Data Centre, UK); inorganics are covered by the Inorganic Crystal Structure Database (ICSD, Gmelin Institute and Fachinformationszentrum Karlsruhe, Germany); biological macromolecules are covered by the Protein Data Bank (PDB, Brookhaven National Laboratory, USA). Consensus among the producers minimizes overlap in areas covered.

Section 2 of this paper outlines the collection criteria, the literature coverage, the scientific editing process, and the details of what an entry contains. Of particular interest to some may be the trends in the complexity and constituents of compounds published over the last 2 decades. The online search, retrieval, and analysis system used for CRYSTMET is illustrated in Sec. 3 and two primary applications of CRYSTMET are described in Sec. 4. Section 5 lists the means by which CRYSTMET is made available and, in conclusion, Sec. 6 sketches two new developments.

## 2. Contents

### 2.1 Inclusion Criteria

Compounds are taken to be metals, henceforth understood to mean metallic elements, alloys, intermetallics or minerals, if they are composed of elements to the left of the Zintl line in the periodic table. Cross-compounds, phases formed with elements immediately to the right of the line, along with some dioxides are included; all other oxides and all compounds containing halides or noble gases are excluded. A fundamental requirement for inclusion in the database is that the composition be clearly defined and that the space group and unit cell be determined.

CRYSTMET, covering the literature exhaustively back to 1913, currently contains about 60 000 entries. Of this number, about half relate to studies in which the positional, thermal, and occupational parameters of all atoms have been determined (refined structures). In the other half, the structure type has either been published by the author or assigned by NRCC/MPDS (assigned structures). For this latter group, in first approximation, the positional and occupational parameters of the structure type may be assumed.

### 2.2 Literature Coverage

**Fig. 1:**
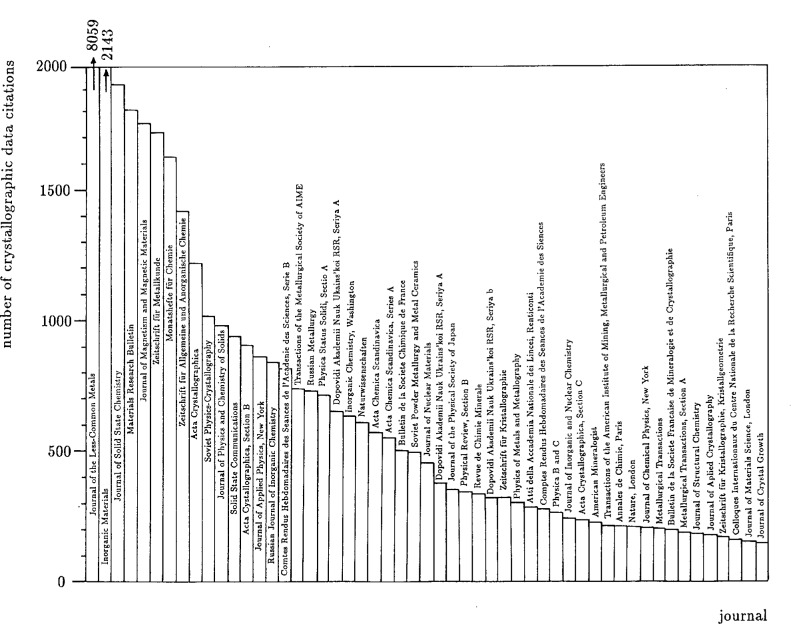
Distribution of the number of crystallographic data citations across key journals.

**Fig. 2:**
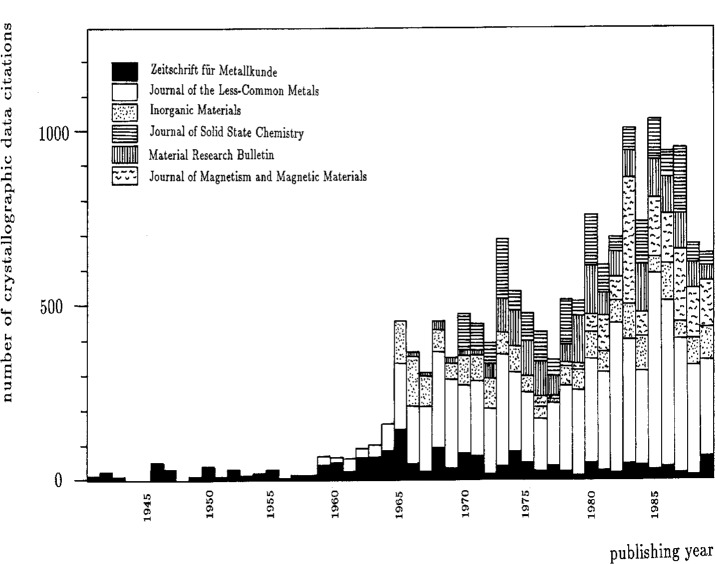
Distribution of the number of crystallographic data citations in the top six journals over the last 50+ years.

Relevant scientific publications are identified through a careful search of the appropriate sections of the following abstract services: Chemical Abstracts, Physics Abstracts, Metals Abstracts, Alloys Index, and Bulletin Signalétique. In addition, over 100 key journals are scanned page by page from cover to cover. Figure 1 shows the distribution of metals crystallographic data entries among the top 50 or so key journals. Figure 2, displaying for the six top journals the number of metals crystallographic data citations as a function of the publishing year, clearly illustrates the increase in the annual number of entries during the last 30 years.

**Fig. 3:**
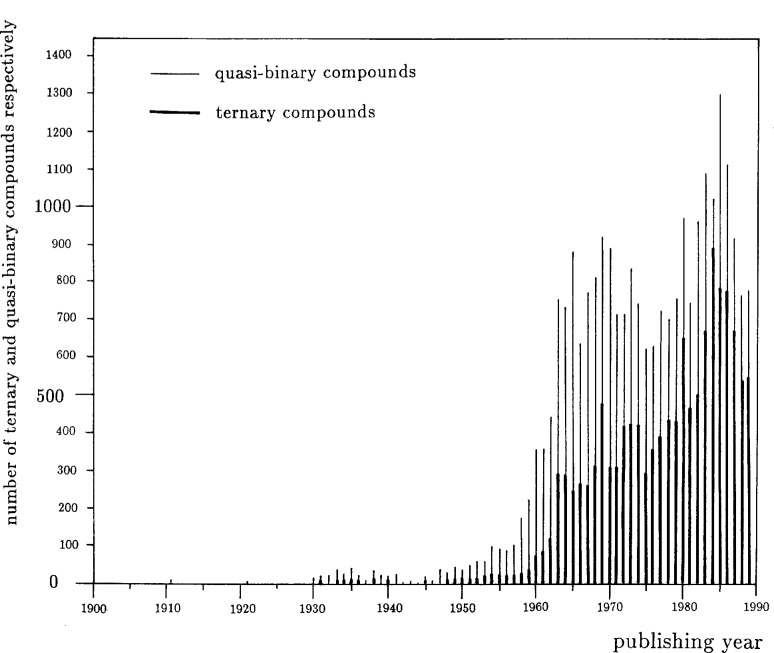
Distribution of the number of ternary and quasi-binary compounds published over the last 90 years.

**Fig. 4:**
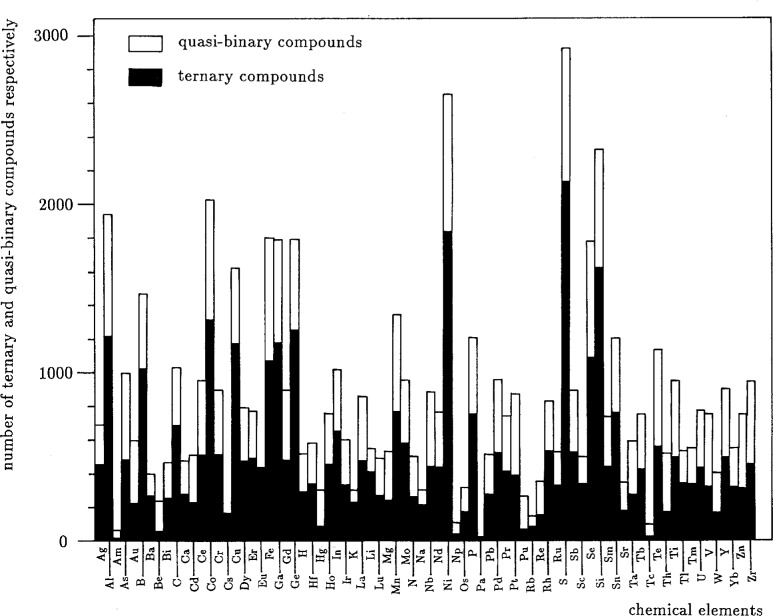
Distribution of the number of ternary and quasi-binary compounds according to the elements they contain.

Some potentially useful intelligence can be uncovered through simple analyses of the studies published. For example, Fig. 3, a plot of the number of ternary and quasi-binary compounds appearing in publications annually, demonstrates a shift in interest over the last 20 years from binary to ternary compounds. Figure 4 reveals that Al, Co, Ni, S, and Se are studied most frequently.

### 2.3 Data Entry

Data are keyboarded directly from a copy of the original publication into a computer file wherein numerous standardization steps and consistency tests are performed.

All crystallographic data are presented in a standard setting [3]. For monoclinic space groups, where there exists more than one standard setting, the one given is that with a unique axis *b*, cell choice 1; for space groups with different possible choices of origin, the one given is that with origin choice 1; for rhombohedral space groups the hexagonal setting is used. Structures are given in the *abc* setting (right-handed) and the first set of coordinates are presented as in [3].

As an additional means of reducing transcription errors, a set of electronic tables such as the following is used to correlate certain information items automatically:

- CODEN—Serial Name
- Space Group Number—Space Group
- Prototype—Fixed Parameter
- Pearson Symbol—Structure Type—Space Group

With the exception of length, which is recorded in Angstroms (Å), SI units are used: kelvins (K) for temperature; gigapascal (GPa) for pressure; degree (°) for angle; and megagrams per cubic meter (Mg/m^3^) for density. Concentration is expressed in atomic per cent (at. %).

### 2.4 Scientific Editing

In those cases where the positional parameters have not been refined or the structure type has not been assigned in the original work, the editors assign a structure type wherever possible. To help ensure correctness of that structure type assignment, the editors also check its position in different correlations through investigations like:

- *c*/*a* − $\bar{R}$ (weighted radii expression) linear dependence;
- location in an iso-stoichiometric chemical element vs chemical element—structure type plot;
- comparison of calculated density (*d_x_*) with the average *d_x_* of all compounds crystallizing in a certain structure type;
- comparison of *c*/*a* values with average *c*/*a* values of all structure types within a Bravais lattice type.

Whenever a structure type has been assigned by an editor, notice is given in the “comments” field.

Before being included in the database, all numeric data are evaluated critically for self-consistency by two independently developed (NRCC and MPDS) special diagnostic programs. It is the structural geometry output from these programs—for example: listings of inter-atomic distances; two- and three-dimensional plots of the structure; atomic environments (coordination polyhedra); nearest neighbour histograms—that reveals problem areas.

Finally, tests such as the following are done to help guarantee the overall accuracy and consistency of other related data items as well as the bibliographic information:

- consistency between Wyckoff notation, site symmetry, fixed and free positional parameters and space group against an *International Tables* [3] file;
- consistency between the publication year and volume against a journal master file;
- consistency between authors’ names and a master file of authors;
- automated detection of spelling errors.

Again, the editors place comments in the “errors” field when they detect an inconsistency within their evaluation procedure or when a representative of a specific structure type behaves significantly different from the average.

### 2.5 Registration

Although the scientific editing catches most duplicate or related compounds, the registration process identifies any residual ones which may have escaped detection. During registration, new entries are compared with existing ones in the database on the basis of:

- chemistry
- cell
- chemistry/cell.

Entries matching on chemistry (formula) indicate that the new study is a similar compound studied under different physical conditions such as high temperature or high pressure.

Those entries which match according to cell imply that the entry is iso-structural with an entry or entries in the database. Additional checks of the structure type and point-sets are carried out to confirm that indication.

Finally, after being subjected to further checks of structure type and point-sets, those entries matching both on formula and cell are noted to have been studied previously.

### 2.6 Components of a Complete Entry

The data items contained in a full entry are listed below. Of course, depending on the type of entry and on what was given in the original work, not all items appear in every entry.

*Number:* At the abstracting stage each entry is assigned a unique serial number for local use.
*Formula:* The chemical formula for each phase is given; in general the format is the element symbol followed by the element count which may be non-integer.
*Structure Type:* This field is composed of two parts—the structure type^2^ and the Pearson symbol^3^ [4].
*Reference:* The journal citation consists of the journal CODEN, volume, page, part (if any) and year. (The CODEN is as given by Chemical Abstracts or, failing that, as assigned by the CRYSTMET editors.)
*Structure Data:* This field consists of several sub-fields used to indicate certain experimental parameters associated with the structure determination of a phase. These sub-fields are: type of specimen studied (powder or single crystal); refined or assigned structure; number of reflections; method of data collection; temperature and pressure values for the data collection; variety of *R*-factors.
*Cell Data:* Included here are the published unit cell parameters (*a*,*b*,*c*,*α*,*β*,*γ*) with their associated standard deviations (when available), the space group symbol in Hermann-Mauguin notation [2], the crystal system and the number of formula units in the unit cell.
*Authors:* The full list of names of all authors is given according to standard conventions.
*Remarks:* Contains comments about the source of the cell data (e.g., crystallographic study or powder pattern).
*Solute:* For solid solution studies the formula range and the associated cell parameters are recorded.
*Atomic Coordinates:* This field contains the *x*,*y*, and *z* coordinates as well as the occupancy and thermal parameters. When atom coordinates are published as fractions, they are coded accordingly; when two or more atoms occupy the same site, an indeterminate atom symbol (e.g., M) is assigned having the coordinates of that site and a note is made in the remarks field (see lines RM and AT in the example following). The recorded temperature factors can take the form of isotropic or anisotropic *U*’s, *B*’s, or *β*’s.
*Density:* The measured density, with standard deviation, is given along with the calculated density and temperature of density measurement.
*Analysis:* This record indicates the percentage composition of the atoms in the phase.

The following two fields are used only for minerals:

*Locality:* the locale at which the mineral was found; if the mineral is prepared synthetically, the locality is recorded as synthetic.
*Phase Name:* the generally accepted name for that mineral.

An entry as it appears online is shown below. Note that the reduced cell (RC) appears only in the online version of CRYSTMET.

ID:45877FO:Pb3 Se5 Sn2ST:Cl Na sc=cF8RC:a=4.2 b=4.2 c=4.2 al=60 be=60 ga=60CD:spgr=Fm-3m spno=225 sys=cubic dx= 7.41 z=.8AC:a=6.078AU:Berolo O, Woolley JCRE:Mater. Res. Bull. (MRBUAC), 3,445, 1968SL:Pb1-xSeSnx, x= 0-0.4, a= 0.6127-0.6078 nm, linear dependence, (information taken from figure)RM:unit cell dimension taken from figure; M= Pb, SnAT:M 4a m-3m 0 0 0 1Se 4b m-3m 1/2 1/2 1/2 1

The abbreviations used are:

ID: Local Identification number of the entry
FO: Chemical formula
ST: Structure Type
  sc: structure code
RC: Reduced Cell—*a*, *b*, and *c* axes; *α*, *β*, and *γ* inter axial angles
CD: Published crystallographic data:
  spgr: Space group symbol
  spno: Space group number
  sys: Crystal system
  *d*x: Calculated density
  *z*: Number of formula units per unit cell
AC: Cell parameters as published by author
AU: Author(s) name(s)
RE: Reference
SL: Solute (formula range and associated cell parameters for solid solutions)
RM: Remarks
AT: Atomic Coordinates

### 2.7 File Organization

For operational convenience, the database is maintained in both archive and online versions. The archive version is in relational tables, currently operating under the ORACLE^4^ database management system. From this configuration, various other formats, for example the flat file version for export to licensees (see Sec. 5.1), can be derived.

The current format for the online version is an indexed file system under which most of the information items appearing in an entry like that shown in Sec. 2.6, along with some that do not, may be searched. More explicitly, the indexes available cover:

- *chemistry* (formula, elements, formula weight)
- *cell parameters* (authors’ and reduced forms, reduced cell volume)
- *structure* (structure type, Pearson symbol)
- *crystal data* (space group, space group number, system, calculated density, number of formula units per unit cell, number of atoms per unit cell, atomic number, centric or acentric space group, disorder, R-factor)
- *bibliographic information* (author(s) names, CODEN, year of publication)
- *powder diffraction* (whether or not an entry has been determined by powder diffraction)

## 3. Online Search, Retrieval, and Analysis System

To be a truly useful tool, a database such as CRYSTMET must not only be searchable but searchable in as many scientifically interesting ways as possible. To that end, CAN/SND has developed a proprietary custom-designed search, retrieval and analysis (SRA) system, known as GENSEARCH, which is used for all the molecular structure databases on the online service. Acclaimed for its easy to learn, intuitive syntax, GENSEARCH is extremely economical in machine time and powerful in the scope of question that can be handled. To convey a sense of the capabilities of GENSEARCH, it is helpful to consider its two major functions: the queries that can be formulated and the analyses that can be executed.

### 3.1 Types of Queries

Queries may be conveniently grouped into three categories: exact match, partial match, and ranges. In an exact match it is required that all the attributes for a particular field match those specified in the query. Thus, the query to *find all binary alloys with the formula Nb Ta* would be entered as *find fo ’Nb Ta’ and ele 2* and the system would return only entries having the formula Nb Ta. If the *ele 2* part of the question were deleted, the system would return entries having formulae of the form Nb Ta X*_n_* where X may represent one or more other elements (e.g., C N Nb Ta; Cu_6_ Nb Se_8_ Ta; Mo Nb Ta)—an example of a partial match.

Range searches can be conducted on both numeric and character fields. Hence, for example, queries for compounds with a density between 14.5 g cm^−3^ and 15.0 g cm^−3^ (or Mg m^−3^) or for entries where the author spellings range from Smith to Smyth would be expressed as: *find dx 14.5.to.15.0* and *find au smith.to.smyth* respectively.

An especially useful application of range searching is the identification of materials using the reduced cell as described in more detail in Sec. 4.1. In this instance, the unit cell parameters of the subject material entered by the user are transformed by the system into the reduced cell and matched against cells in the database according to a tolerance or range parameter.

### 3.2 Analyses

While many users may prefer to download the information obtained from a given search and analyze it locally, basic analysis tools like FREQuency, MATRIX, and TRANSlate are provided.

The FREQuency command displays, for a given set of hits, the number of entries having each search value within a specified field. It is most easily understood with the use of an example. Issuing the following ‘FIND’ command would retrieve entries that are in the monoclinic crystal system and that have acentric structures. The ’FREQ’ command may then be employed to organize the hits obtained in the search by space group.

FIND sys m and ce a

Set number 1 created with 302 hits. Ready>

FREQ 1 spgr* * * FIND SYS M AND CE A * * *B11b9B1121B21B2^1^2Cc94....P26P2^1^61

The MATRIX command allows one to invert, multiply, transpose, or otherwise manipulate 3 × 3 matrices; the TRANSlate command allows one to transform cell parameters and calculate derivative cells. In both cases, the system prompts for the information required.

## 4. Applications

Two major applications of CRYSTMET involve the identification of unknown materials and the prediction of properties of materials.

### 4.1 Identification Using Cells

The input cell for the unknown material is first reduced and this reduced cell is matched against the database. As mentioned in Sec. 3.1, it is possible to vary the tolerance for matching purposes. In this example it is assumed one has a material which is F-centred cubic with the “a” axis equal to 6.078 Å and that a chemical analysis has shown that the material has only Pb, Sn, and Se present. Invoking the CELLS command, the user enters the cell parameters as prompted and the system responds as shown in Table 1. A search of the database with this reduced cell yields 324 hits.

Exploiting any knowledge of chemistry of the unknown material, in this case a search of the database for all materials which contain only the three elements known to be present, results in just three hits. Combining the results of these two searches using the EVALuate command yields all entries which have similar unit cells and chemistry. In this instance, two entries are found, one of which is the example shown in Sec. 2.6.

### 4.2 Property Prediction

One of the major challenges to the solid-state chemist is the prediction of the properties of a material given its chemical formula. Innovative exploitation of the information contained in CRYSTMET offers new avenues of approach.

As an example of how poorly conventional methodology performs in this area, one may consider the following question. Was it possible to predict the crystal structure and properties of YBa_2_ Cu_3_ O_7_ and Nd_2_ Fe_14_ B before they were synthesized? The structure of the first material could possibly have been anticipated from knowledge of oxide chemistry but the fact that it is superconducting at 95 K could not. For the second material probably neither its structure nor its interesting magnetic properties could have been predicted. This point was emphasized by Cheetham [5] where he observed that, in many of the systems studied to date, discoveries have arisen more from serendipity or perservance than from rational design.

**Fig. 5:**
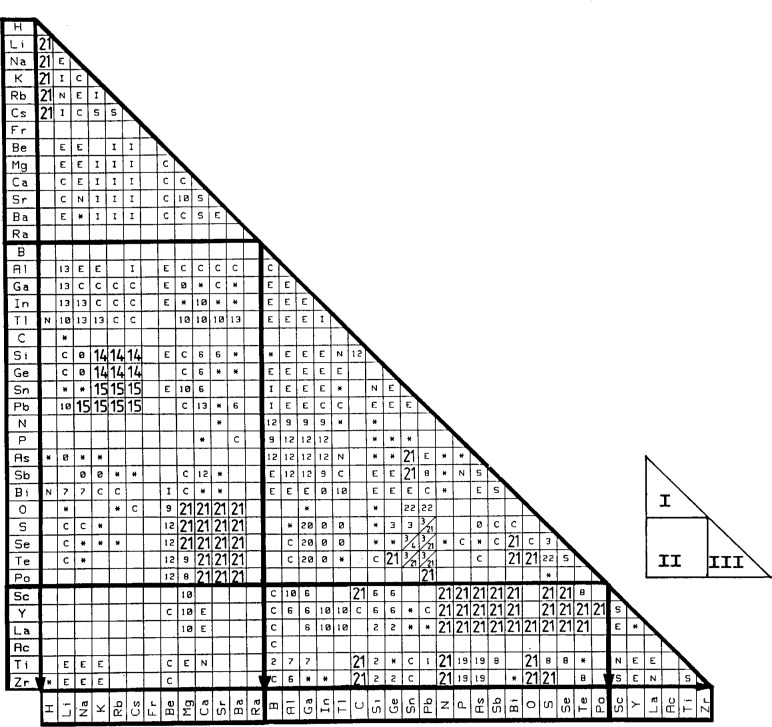
Part of a chemical element vs chemical element structure type map for AB compounds. Legend:
1AuCd6BCr11FeSi16CW21ClNa2BFe7AuCu12SZn(*cF*8)17HgS22PbO3GeS8AsN13NaTb18CoSn23LiPd4MnP9SZn(*hP*4)14GeK19AsTi24AlCe5AlEr10ClCs15NaPb20GaS25CrFe Ø —structure with less than three representatives I —non-compound forming system of the complete insolubility type S —non-compound forming system of the complete solubility type E —non-compound forming system of the simple eutectic-peritectic type N—non-compound forming system without nearer specification * —compound existence but no knowledge of crystal structure C —phase diagram has established non-existence of AB compounds / —existence of two modifications Note: These results are taken from Ref. [6] and reflect the existence of crystal structures at that time.

A better methodology is to organize and classify creatively the property and structure of known systems to assist in the quest for those of others. Structure/property maps are one effective tool. Such maps are two- or three-dimensional maps which employ a variety of coordinate systems to arrange the data. Some of these indices are crystal radii, electronegativity, principal quantum number, heats of reaction, transition temperature (*T*_m_), atomic number and others. Based on information contained in CRYSTMET, Fig. 5 is a chemical element vs chemical element map for AB compounds showing the distribution of the structure types based on results taken from Ref. [6]. One can utilize such a map to predict as well as to check structure types. A full survey of the use and application of structure maps is given by Burdett and Rodgers in the *Encyclopedia of Inorganic Chemistry* [7].

## 5. Availability

Access to CRYSTMET may be obtained as indicated in the following three sections.

### 5.1 Licensed Copy

Those with adequate storage facilities and an appropriate database management system may choose to obtain a copy of the CRYSTMET data for individual, institutional, or geographically restricted online use. Once the formal license agreement is completed and the annual fee paid, the client is supplied a flat file copy of the database either on magnetic media or via file transfer. No SRA software is supplied.

### 5.2 Online Service

To take advantage of GENSEARCH, users must access CRYSTMET via the online service offered by CAN/SND. With the rapid development of the Internet, interactive communication is now most easily and economically achieved by *telnet*ing to the CAN/SND server; the X.25 packet-switched networks (DATAPAC in Canada, TELENET or TYMNET in the USA, etc.) provide a lower speed alternative. In either case, no long distance telephone calls are involved. Files of results created during a session may be transferred readily via e-mail, using the SEND command, or via ftp to the user’s computer for subsequent perusal or analysis.

For CRYSTMET, two online fee schedules are available. One may choose a rate proportional to usage which includes telecommunication, connect time and cpu usage costs (plus a small annual fee) or one may choose an annual flat fee with unlimited usage but restricted to access only via the Internet. This latter option should be especially attractive to those who use CRYSTMET heavily enough to consider licensing a copy but who have no local SRA capabilities comparable to GENSEARCH.

### 5.3 Customized Search

This is a service whereby CAN/SND staff execute searches and analyses of CRYSTMET, or any other database, on behalf of a client for a fee. Such clients tend to be those who (1) do not have access to a computer terminal or communication facilities or who (2) do not use the system sufficiently often to justify the overhead in learning and re-learning the various protocols and procedures.

## 6. Current Plans

Efforts are ongoing on several fronts to ensure that CRYSTMET continues to evolve into much more than a compilation of entries containing factual information and crystallographic data. The following sections briefly describes two of those endeavours.

### 6.1 WWW Browser capability

A prototype search and retrieval system, which may ultimately supersede GENSEARCH, has been developed. Like GENSEARCH, the new system will function, not only with CRYSTMET, but with the other structural databases. Combining the powerful search possibilities of a relational database management system like ORACLE with an interface based upon a modern browsing tool like Netscape, the new system promises not only to be extremely flexible and easy to use but virtually free of the frustrations, ensuing from the idiosyncracies of various system and terminal emulations, which often arise when connected remotely to different hosts.

### 6.2 Dynamically Accessible Structure/Property Maps

CAN/SND feels it is critical that databases such as CRYSTMET be viewed as much more than a handbook through which one may flip electronically, seeing or learning no more than the authors and publishers had the foresight to provide via the indexes created. One way of encouraging users to view the data as a source of new insights is to furnish appropriate tools such as online structure/property maps.

Thus, coming down the development pipeline is a mechanism whereby a user would be able to perform online some of the activities described in Sec. 4.2 without having to be an expert in the field. Hence, one could generate structure/property maps dynamically from a selection of indices (e.g., atomic number vs atomic number for two elements A*_m_*B*_n_*, crystal radii versus electronegativity, etc.) and parameters (structure type, density, crystal system, etc.). Clicking on a specific point or area on such a map (similar to Fig. 5) would then, via a hypertext link, generate a plot of the relevant structure type or search the database for details of pertinent entries.

## Figures and Tables

**Table 1:** Response to CELLS command

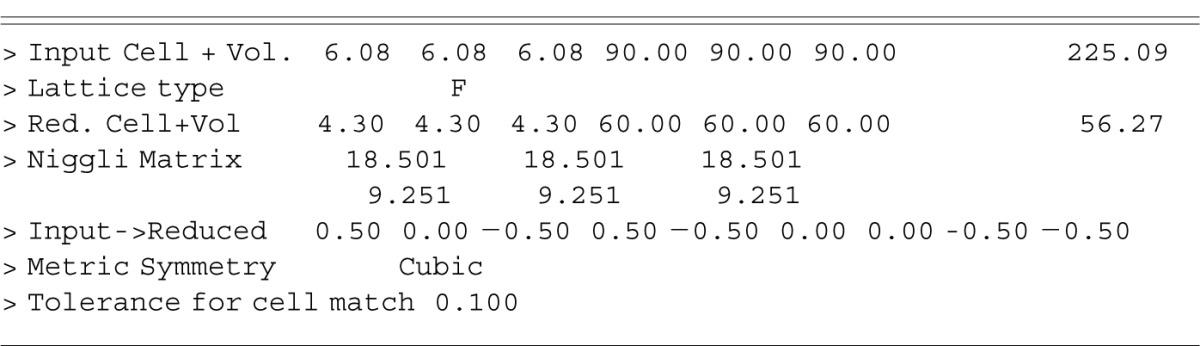

## Footnotes

2 The assigned structure type will be the formula of the first published structure with the same space group, occupied sitesets and atomic coordination. For example, Mg_9_ Pd_11_ and Au Cu are isostructural or isotypic, each has the space group P4/mmm and occupied sitesets 1(a), 1(c), and 2(e).

3 The Pearson symbol is composed of three parts: the first is a lowercase letter defining the crystal system (a,m,o,t,h or c); the second is a capital letter defining the lattice centring (P,C,F,I or R); the third is the number of atoms of the phase in the unit cell. (For monoclinic and orthorhombic systems, lattice-centring of the type A,B,C are denoted as C.) In the examples of footnote 2, the derived Pearson Symbol is tP4.

4 Certain commercial equipment, instruments, or materials are identified in this paper to foster understanding. Such identification does not imply recommendation or endorsement by the National Institute of Standards and Technology, nor does it imply that the materials or equipment identified are necessarily the best available for the purpose.
